# Annotations

**Published:** 1902-06-21

**Authors:** 


### Annotations.

### Fasting.

As a season is approaching which will undoubtedly be one involving many feasts, let us consider their antithesis, and take fasting as our theme. The ancients, as the saying goes, "knew a thing or two," and one need hardly be surprised that at a period when self-indulgence took its grosser forms, and when gluttony was the most popular of vices, those kindly saints who, though "dead on" heretics, did so much to encourage true believers in the paths of virtue, should have invented fasting as a duty and a penance. As a weekly recurring duty fasting undoubtedly became a health-giving ordinance, while it had this great superiority over other forms of penance, that it generally left the penitent in better form than it found him. And therein lies its virtue now. We all eat more than we require, and this daily repeated superfluity lends to stodginess. In a more primitive state of society meals were more irregular, and the amount of food tallied more with the effort expended in obtaining it. Now we eat because it is a meal time; too many of us eat not by rule but to repletion; while probably all of us eat again before we are really hungry. Day after day a little more is taken than is used, and this excess either disturbs the liver, or teases the stomach, or circulating in a hyperplastic blood, leads to torpor, or sometimes is put by—out of harm's way for the time, but much to the distress of the patient later on—in the form of fat. Thus we never have an opportunity of striking a proper balance between intake and output unless we follow the wise maxims of the Church, and fast once a week, not merely abstaining from the more toothsome delicacies, but fasting honestly, even to emptiness and discomfort. Let us remember that, speaking now not of invalids but of the healthy, there is probably no one who has not stored up in the various corners of his frame, sufficient food to keep him going, not comfortably, perhaps, but well, for at least two or three days, and that it is good for us to cultivate the habit of living on our supplies rather than trusting to a hand-to-mouth existence.

### The British Medical Association.

At a statutory meeting held on Wednesday, at Exeter Hall, of the British Medical Association a resolution was carried by the necessary majority, namely, three to one, in favour of accepting the new articles of association which have been prepared under the direction of a committee appointed for the purpose by the Council in pursuance of resolutions passed at the annual general meeting of the Association at Cheltenham in 1901. The voting was 96 for and 28 against the resolution. The new departure is rather a large venture, and we can but hope that an association which has done much good work for the profession in the past will continue to do so under the new *régime*, as it doubtless will, should its management fall into good hands. The strength of the Association has always lain in the difficulty which has existed in effecting any rapid change in the constitution of the Council in response to sudden changes in public sentiment. Coupled as this has been with an obstinate determination to take no notice of the wayward gusts of opinion expressed by annual meetings, the Council has stood firm and has managed the business of the Association with marked financial success. All this refusal to carry out the wishes of the general meetings has, however, been shown to be illegal, so it has been found necessary to draw up this new constitution. The future of the Association will, of course, depend, as its past has depended, on the successful conduct of its Journal, the fount from which the money flows; but much will also hinge upon the degree of interest taken by individual members in the meetings of those small bodies the "divisions" into which the Association is to be cut up. Unless the members can be induced to take an interest in its affairs the whole Association will fall into the hands of a few wirepullers.

### Our Sham Civilisation.

At the last meeting of the Epidemiological Society a case was discussed in which there had been considerable difficulty in arriving at a satisfactory diagnosis. The case was supposed to have been one of hæmorrhagic small-pox; but there was a suspicion that it might have been one of plague, and even after *post-mortem* examination, and a complete bacteriological investigation, it was by no means clear that either of these diseases had been present; indeed there was much to point to the probability that the case was one of virulent septic infection due to the frightfully insanitary conditions of the house in which the patient (a nurse) had been working; and it is to the evidence given of the existence of such insanitary conditions in the very centre of London, notwithstanding all our sanitary machinery, that we would more particularly direct attention. The patient, who was a nurse from a home near the Strand, had for the three weeks preceding her illness been nursing the proprietor of a restaurant in an adjoining street, who was suffering from acute rheumatism, and it is stated that she, together with the other nurse attending the case, had slept and taken her meals at the home, since there was no accommodation at the house of the patient, this house being in such an insanitary condition that neither they nor the cooks could eat on the premises, which were swarming with rats. Now we cannot help thinking that there are a good many people who would much like to know which is the particular "restaurant in an adjoining street near the Strand" the premises of which are in such an insanitary condition that neither the nurses nor the cooks could eat there, which are swarming with rats, and the state of which is such as to have given rise in the minds of the learned men who discussed this case to the idea that the cause of this unfortunate nurse's death may have been a septic disease arising from "the highly insanitary condition of the house." To the tune of drums and trumpets representatives of the Empire will march next week in Coronation Procession down this very Strand; an assembly gathered from every quarter of the globe to give due honour to their sovereign, and also to see this great London of ours, this capital of a great civilising, sanitation-teaching country. And this is what we show them—dwellings unfit for habitation, yet inhabited in defiance of all our laws; buildings in which not even the cooks will eat, yet used as a public restaurant!

